# Effect of ultraviolet light treatment on surface hydrophilicity and human gingival fibroblast response on nanostructured titanium surfaces

**DOI:** 10.1002/cre2.108

**Published:** 2018-06-11

**Authors:** Nagat Areid, Ari Peltola, Ilkka Kangasniemi, Ahmed Ballo, Timo O. Närhi

**Affiliations:** ^1^ Department of Prosthetic Dentistry, Stomatognathic Physiology University of Turku Turku Finland; ^2^ Turku Clinical Biomaterials Centre University of Turku Turku Finland; ^3^ Division of Prosthodontics and Dental Geriatrics, Department of Oral Health Sciences, Faculty of Dentistry University of British Columbia BC Canada; ^4^ Department of Oral and Maxillofacial Disease Turku University Hospital Turku Finland

**Keywords:** contact angle, fibroblast, nanostructure, TiO2, UV light

## Abstract

This study was designed to investigate the effect of nanostructured TiO_2_ coatings on human gingival fibroblast and to explore the influence of ultraviolet (UV) light on surface wettability and cellular response. Ti‐6Al‐4V titanium alloy discs (*n* = 96) were divided into three groups: a sol–gel‐derived MetAlive™ (MA) coating; hydrothermal (HT) coating; and a non‐coated (NC) group. Forty‐eight titanium substrates were further treated with UV light for 15 min. The water contact angles of the substrates were measured using the sessile drop method. Human gingival fibroblasts were used to evaluate the cell adhesion strength and cell proliferation on experimental surfaces. The strength of cell adhesion against enzymatic detachment was studied after 6 hr of adhesion using gentle trypsinization for 15 min at room temperature. A fluorescence microscope was used for cell imaging (Zeiss‐stereo‐lumar‐v12), and images were analyzed for cell counting, and the percentage of detached cells were calculated. The proliferation of cultured cells up to 10 days was determined according to the cell activity using Alamar Blue™assay. The HT group had the lowest contact angle value (31.1°) followed by MetAlive™ (35.3°), whereas the NC group had the highest contact angle (50.3°). After UV light treatment, all surfaces become considerably more hydrophilic. There was a significant difference in the amount of adherent cells between sol–gel and HT groups when compared with the NC group (*p* < .05) with detachment percentages of 35.8%, 36.4%, and 70.7%, respectively. All substrate types showed an increase in cell proliferation rate until 10 days. It can be concluded that nanostructured titanium oxide implant surfaces, obtained by sol–gel and HT coating methods, enhance the surface wettability and improve human gingival fibroblast function in terms of adhesion and proliferation rate when compared with non‐coated surfaces. UV light treatment clearly enhances the wettability of all titanium surfaces.

## 1 INTRODUCTION

Implant‐supported dental prostheses have provided success rates exceeding 90% over the last several years (Chuang, Tian, Wei, & Dodson, 2002). Successful implantation depends not only on osseointegration but also on the peri‐implant soft tissue attachment around the transmucosal area of the implant. Modification of implant surfaces may allow faster integration after implantation and enhance long‐term bone maintenance (Coelho et al., 2009; Daugaard, Elmengaard, Bechtold, & Soballe, 2008). The surface modification of the transmucosal area significantly affects soft tissue attachment (Hoshi, Negishi, Okada, Nonami, & Kimoto, 2010; Werner et al., 2009), the impediment of bacterial biofilm adhesion (Frojd et al., 2011), and the preservation of the crestal bone of the alveolar ridge (Botos, Yousef, Zweig, Flinton, & Weiner, 2011).

A protective soft tissue seal between the oral environment and the underlying peri‐implant bone is considered important for the protection of the tissue–implant interface from bacterial invasion, which may lead to unwanted clinical complications, such as inflammation, marginal bone resorption and soft tissue recession. This soft tissue seal between the transmucosal implant surface and the surrounding soft tissue is identified as a fibroblast‐rich tissue, which is the predominant barrier to bacterial invasion into peri‐implant tissues. Therefore, the formation of a proper abutment/soft tissue seal is believed to be an important factor for the long‐term success of implant therapy (Abrahamsson, Berglundh, Glantz, & Lindhe, 1998). Different surface treatments are routinely used to increase implant roughness and promote osseointegration (Mendonca, Mendonca, Aragao, & Cooper, 2008), whereas the transmucosal portion of the implant surface is usually made of a smooth turned titanium surface to reduce bacterial adhesion and biofilm formation. However, there is good evidence that implant surface modification may facilitate peri‐implant soft tissue attachment (Schupbach & Glauser, 2007; Welander, Abrahamsson, & Berglundh, 2008).

The modification of the implant surface topography at nanoscale level has been largely investigated. Surface nanostructure can alter the surface chemistry and topography, which may favorably influence molecular and cellular events and promote bone‐bonding behavior at the titanium–bone interface (Kubo et al., 2009; Wennerberg & Albrektsson, 2010), whereas little is known about their effect on soft peri‐implant tissues.

Titanium and titanium alloys, such as Ti‐6Al‐4V, are fairly good biomaterials and have been widely used in dentistry and orthopedics because of their superior biocompatibility and mechanical properties. Titanium surfaces react with oxygen when exposed to air or water and form a thin film (4–6 nm) of protective oxide. This native nanocrystalline titanium dioxide film results in superior corrosion resistance and good biocompatibility (Liu, Chu, & Ding, 2004). However, this film is usually non‐uniform, mechanically weak, and does not enhance the wound healing process (Liu et al., 2004). Therefore, various advanced modification techniques have been applied to improve the properties required to enhance bone integration; for example, bioactivity and bactericidal properties. These techniques include anodic oxidation (Jimbo et al., 2008), sol–gel coating method (Peltola, Patsi, Rahiala, Kangasniemi, & Yli‐Urpo, 1998), and chemical or hydrothermal (HT) methods (Kim, Miyaji, Kokubo, & Nakamura, 1996). However, the sol–gel method does not allow for coating of complex objects, and anodizing improves biological properties only to a limited degree. In contrast, the HT method appears to be a relatively simple and more feasible chemical coating method. Hamad et al. (2002) reported that HT treatment in a CaO solution increased the precipitation of apatite on the titanium surface (Hamad et al., 2002). A study by Nakagawa, Zhang, Udoh, Matsuya, and Ishikawa (2005) suggested that Ti—O—Ca bonding formed by HT treatment in a CaCl_2_ solution was a predictable way to improve the bioactivity and osteoconductivity of titanium implants. HT‐treated TiN coating in CaCl₂ solution improved wettability and promoted fibroblast adhesion and proliferation as shown by Shi, Xu, Munar, and Ishikawa (2015).

Photocatalysis of TiO_2_ has been investigated extensively during the past 20 years. The actual discovery of this technology came with TiO_2_ coatings on solid surfaces, providing self‐cleaning, self‐sterilizing and more recently antibacterial functions based on the photo‐induced hydrophilicity and decomposition reaction (Fujishima, Zhang, & Tryk, 2008). When anatase TiO₂ is irradiated with ultraviolet (UV) light having a wavelength shorter than 385 nm, an electron–hole pair is generated. The adsorbed molecules such as oxygen and water will rapidly be reduced and oxidized to produce superoxide ions (O₂^−^) and hydroxyl radicals (OH^−^), respectively. These can react with organic material, such as adherent bacteria and mineralizing them into CO₂ and H₂O (Fujishima et al., 2008; Riley, Bavastrello, Covani, Barone, & Nicolini, 2005).

UV photofunctionalization of titanium has drawn considerable attention recently as a surface modification method for titanium surfaces to enhance biologic capacity and physicochemical properties (Aita et al., 2009; Iwasa et al., 2010). “The biomechanical strength of *in vivo* osseointegration for UV‐treated implants was three times greater than that for untreated implants at the early healing stage in an animal model, and nearly 100% bone to implant contact (BIC) was achieved, as opposed to less than 55% BIC for untreated implants” (Aita et al., 2009). The UV photofunctionalization is expected to be a new effective and simple approach on various titanium surfaces without altering the existing topography, roughness, or other morphologic features of the implants (Aita et al., 2009). Photocatalytic TiO_2_ films have been shown to be multifunctionally effective in biomedical applications due to their superhydrophilic and bactericidal properties, both induced by UV illumination (Rupp et al., 2010). The possibility of adding an in situ self‐cleaning and antibacterial feature to biomedical implants and devices where UV light can access, using a simple method, could successfully help to reduce implant infection related complications.

The aim of this study was to investigate the effect of nanostructured TiO_2_ coatings on human gingival fibroblast. Furthermore, this study aimed to explore the effect of UV light on the biologic and physicochemical properties of these surfaces.

## 2 MATERIALS AND METHODS

### 2.1 Sample preparation

Ti‐6Al‐4V (grade) titanium alloy discs (diameter 7 mm and thickness 1 mm) were prepared for the study. The discs were ground with silicon carbide paper of 1,200 grit with Ra value of 0.15 μm, cleaned ultrasonically in acetone and ethanol (5 + 5 min), and dried in air before performing surface treatments.

### 2.2 Surface treatments

The discs were divided into three groups and provided with the following surface treatments: The first group was coated with a sol–gel‐derived MetAlive™ (MA) coating and served as a positive control group; the second group was treated with the HT method; and the third group was left untreated (NC) and served as Negative control group. All specimens were rinsed with acetone for 5 min and then in ethanol for 5 min, followed by thorough drying before testing.

#### 2.2.1 Sol–gel coating preparation

The nanoporous TiO_2_ thin film was prepared on the titanium substrate using a sol–gel treatment. The sol was made as originally described by Peltola et al. (1998) and Jokinen et al. (1998). In short, solution I contained commercially available tetra isopropyl orthotitanate, Ti {OCH(CH_3_)_2_}_4_, and was dissolved in absolute ethanol (solution I). Ethyleneglycol monoethylether (CH_3_CH_2_OCH_2_CH_2_OH) deionized water and fuming hydrochloric acid (HCl 37%) were dissolved in ethanol (solution II). Solutions I and II were mixed rapidly and stirred effectively for 3 min. The sol was kept at 0 °C during aging and the dip‐coating process. The coating procedure started after 24 hr of sol aging, and samples were coated with five layers. After deposition of the layer, the substrates were sintered at 500 °C for 10 min; the coatings were cleaned ultrasonically in acetone for 5 min, in ethanol for 5 min, and finally dried at the ambient temperature.

#### 2.2.2 HT treatment

After ultrasonic washing of the titanium substrate with ethanol and distilled water for 5 min each, respectively, a HT suspension was first prepared using reagent grade chemicals. This was done by dissolving Titanium oxide (TiO_2_), purified water, and 1:10 diluted tetramethylammonium hydroxide (N(CH_3_)_4_^+^ OH)^−^ and mixed for 5 min. Titanium plates were laid at the bottom of Teflon containers, which consisted of a Teflon inner vessel and a stainless‐steel jacket; within this, the HT suspension was added. Then, the vessel was kept at 150 ± 10 °C in a constant temperature oven for 48 hr. After the HT treatment period, the titanium plates were removed from the vessel and cooled in air. All the plates were washed with distilled water in an ultrasonic bath for 10 min.

### 2.3 UV light treatment

Forty‐eight substrates of the three different groups (MA, HT, and NC), *n* = 16 for each group, were treated with UV light for 15 min under ambient conditions using a 36 W puritec HNS germicidal ultraviolet lamp (Osram GmbH; Germany), with dominant wavelength of 254 nm. These UV‐treated titanium substrates were used immediately (fresh surface) for the analysis of surface properties and cellular response and were compared with the non‐UV‐treated ones.

### 2.4 Contact angle measurements

The water contact angle of each sample was measured using the sessile drop method (described by de Jong van Pelt, & Arends in 1982) with a contact angle meter (KSVCAM100 KSV, Instrument LTD, Finland). The contact angles were determined by placing four drops of distilled water on each specimen, at room temperature. A drop was deposited on the surface of the specimen and imaged for 20 s by collecting one image per 2 s, 10 images per each drop; the result was the mean of at least 40 images on each specimen.

### 2.5 Cell cultures

Human gingival fibroblasts used in this study were obtained, after informed consent, from patients undergoing tooth extractions at the Institute of Dentistry, University of Turku as explained earlier (Oksanen & Hormia, 2002). Shortly, tissue samples from periodontally healthy sites were used for explant cultures. Cells were cultured in Dulbecco's modified Eagle's medium supplemented with 10% fetal bovine serum and antibiotics (all from Gibco BRL, Life Technologies, UK). Culture medium was changed three times a week. Human gingival fibroblasts were used to evaluate the cell adhesion resistance and cell proliferation on experimental surfaces.

### 2.6 Cell adhesion resistance against enzymatic detachment

Fibroblasts were plated on UV and non‐UV treated titanium substrates at a density of 20,000 cells/cm^2^ (38,000 cells/well) and allowed to adhere for 6 hr at 37 °C. Seventy‐two titanium substrates were used. Each of the experimental groups (HT, MA, and NC) had 24 substrates. Half of the substrates in each group (*n* = 12) were UV‐treated, whereas the remaining 12 were left without UV treatment. In order to determine detachment rate, two 24‐well plates were used (one for the non‐trypsinized samples and one for the trypsinized samples), six parallel samples were used, and the experiment was repeated twice. The resistance against enzymatic detachment was evaluated by trypsinizations with 1:10 diluted enzymes in phosphate‐buffered saline (PBS; 0.005% Trypsin, 0.05 mM ethylenediaminetetraacetic acid [EDTA]; Gibco, Invitrogen). A 1:500 trypsin concentration was used for 15 min. The method was modified from Anselme et al. (2000) and Meretoja, Rossi, Peltola, Pelliniemi, and Narhi (2010). Substrates were washed three times with PBS to remove non‐adherent cells and placed on clean culture plates with enzyme solution (1.25 ml per substrate). Trypsinized plates were incubated on a rotary shaker (Max Q 2000; Barnstead International, Iowa), 100 rpm at room temperature for 15 min. Trypsin was removed, and Dulbecco's modified Eagle's medium was placed. Substrates were washed three times with PBS, and cells were fixed with formalin for 15 min and washed three times with PBS. Cells were stained with a fluorescence stain (Hoechst 33342). Thereafter, the substrates were incubated on a rotary shaker (100 rpm at room temperature) for 15 min after which the substrates were washed three times with PBS. A fluorescence microscope was used for cell imaging (Zeiss‐stereo‐lumar‐v12), with an objective lens of NeoLumar 0.8×, for both trypsinized and non‐trypsinized samples, and images were analyzed for cell counting, and the percentage of detached cells were calculated. Then, all substrates were rinsed in PBS and dried in increasing ethanol series, and then, the final 100% ethanol wash was replaced with hexamethyldisilazane (HMDS) 50% for 30 min, followed by 100% HDMS overnight. An approximately 20‐nm‐thick gold layer was applied on samples with a sputter coater for examination in a scanning electron microscopy (SEM; Phenom ProX‐Netherlands).

SEM images with different magnifications were collected for trypsinized and non‐trypsinized groups.

### 2.7 Cell proliferation

Human gingival fibroblasts were plated at a density of 20,000 cells/cm^2^on the titanium substrates and cultured for up to 10 days. The proliferation of cultured cells was determined according to the cell activity using Alamar Blue™assay. The titanium substrates (*n* = 24), four substrates for each UV and non‐UV group, were withdrawn from the culture, at predetermined times (Days 1, 3, 7, and 10) and placed into sterile culture plates containing fresh culture medium with 10% assay reagent. After 3 hr of incubation, the absorbance value was read at 570 and 595 nm using an ELISA plate reader, and the cell proliferation rate was measured at different time points. Then, the substrates were rinsed three times with PBS and fixed with 2.5% glutaraldehyde. All substrates were rinsed with PBS and dried through a series of graded alcohol and sputter‐coated with gold. Finally, the cell morphology was observed with SEM.

### 2.8 Scanning electron microscopy

The surface topography of the substrates was characterized using field‐emission SEM. An approximately 20‐nm‐thick gold layer was applied on samples with a sputter coater, and secondary electron images were recorded with SEM. All the tested substrates were fixed with 2.5% glutaraldehyde solution and were rinsed in PBS and dehydrated at increasing alcohol concentrations series (35%, 50%, 70%, 95%, and 100%) for 30 min each; then, the final 100% ethanol wash was replaced with HMDS 50% for 30 min, followed by 100% HDMS overnight. The samples were stored in the desiccator for 24 hr, before use for SEM analysis. An approximately 20‐nm‐thick gold layer was applied on samples with a sputter coater for examination in an SEM (Phenom ProX‐Netherlands).

### 2.9 Statistical analysis

Statistical analysis was performed with the IBM SPSS v.23.0 software package (IBM SPSS Inc.). To analyze the differences among several means, the data were analyzed with one‐way analysis of variance followed by Tukey's post hoc test. Differences were considered significant at 95% confidence level, with *p* values below .05 (^*^ *p* < .05; ^**^ *p* < .01; ^***^ *p* < .001).

## 3 RESULTS

### 3.1 Contact angle

**Figure 1:**
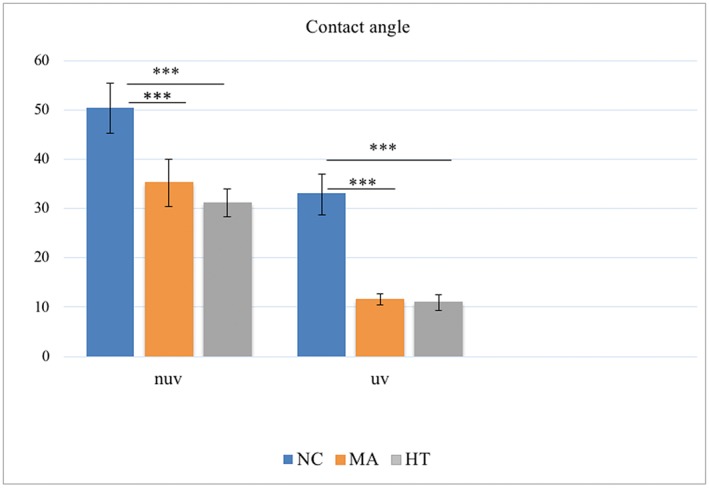
The contact angle values of distilled water obtained by the sessile drop method on different surfaces before ultraviolet (NUV) and after (UV) light treatment. Statistically significant differences were found between the hydrothermal (HT) and MetAlive™ (MA) coating groups and the non‐coated (NC) group. (^***^ *p* < .001)

The HT group had the lowest water contact angle (31.1°) followed by MA (35.3°), whereas the NC group had the highest contact angle value (50.3°). The UV light treatment significantly enhanced substrates' hydrophilicity: After UV treatment, the water contact angles dropped for all substrates, being 10.9°, 11.5°, and 32.9° for HT, MA, and NC, respectively (Figure 1).

Although there was no significant difference between the HT and MA UV‐treated groups, their contact angles were significantly lower than that of the NC UV group.

### 3.2 Cell adhesion resistance against enzymatic detachment

**Figure 2:**
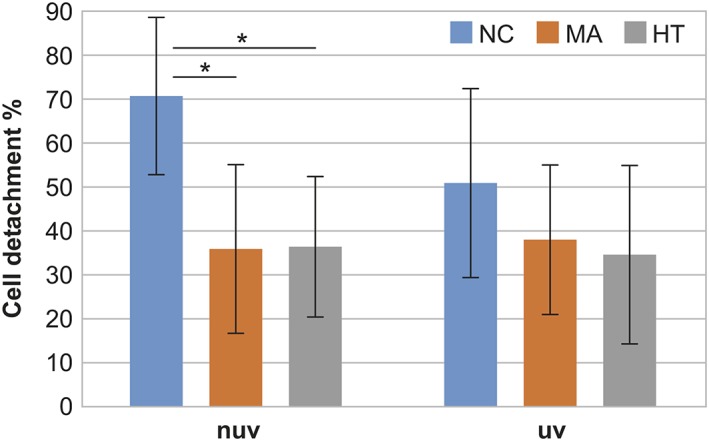
The cumulative amounts of fibroblast detached from the MetAlive™ (MA) coating, hydrothermal (HT) TiO_2_, and non‐coated (NC) titanium surfaces after 15 min of gentle trypsinization. Statistically significant differences in the amount of adherent cells were found between MA and HT groups when compared with the NC group (^*^ *p* < .05), with detachment percentages of 35.8%, 36.4%, and 70.7%, respectively. NUV = non‐ultraviolet; UV = ultraviolet

Human gingival fibroblasts were incubated for 6 hr on titanium substrates, and no statistically significant difference in the amount of adhered cells among the substrate types was found (*p* ˃ .05). The strength of cell adhesion against enzymatic detachment was studied after 6 hr of adhesion using gentle trypsinization for 15 min at room temperature. There was a significant difference in the amount of adherent cells between sol–gel and HT groups when compared with the NC group (*p* = .039, .049), respectively, with detachment percentages of 35.8%, 36.4%, and 70.7% respectively (Figure 2). However, no significant difference in cell adhesion resistance between the UV‐treated and non‐UV‐treated groups was observed.

### 3.3 Cell proliferation

**Figure 3:**
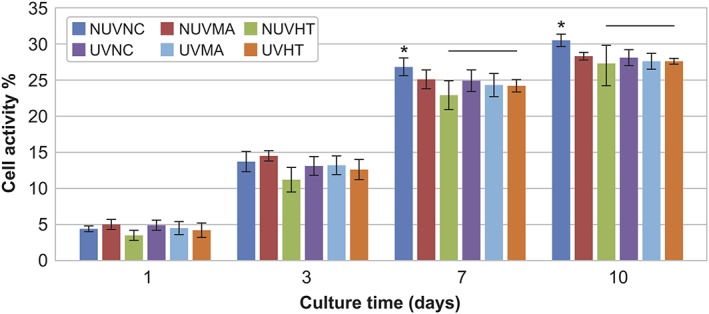
Proliferation rate of human gingival fibroblasts on the substrate investigated with or without ultraviolet (UV) light treatment. All substrate types showed an increase in cell activity with time up to 10 days. Statistically significant difference in cell activity were found among the non‐UV (NUV)‐treated substrates at all times (^*^ *p* < .05). After UV light treatment, all the substrate types showed an increase in proliferation rate throughout the observation period. HT = hydrothermal; MA = MetAlive™; NC = non‐coated

Human gingival fibroblasts were cultured for 10 days on the titanium substrates. All substrate types showed an increase in cell activity with time (Figure 3). There was a significant difference in cell activity among the non‐UV‐treated substrates at all times (*p* < .05); the HT group showed lower proliferation rate, whereas the NC group showed the highest proliferation rate at Days 7 and 10. After UV light treatment, all the substrate types showed an increase in proliferation rate throughout the observation period.

### 3.4 Microscopy

**Figure 4:**
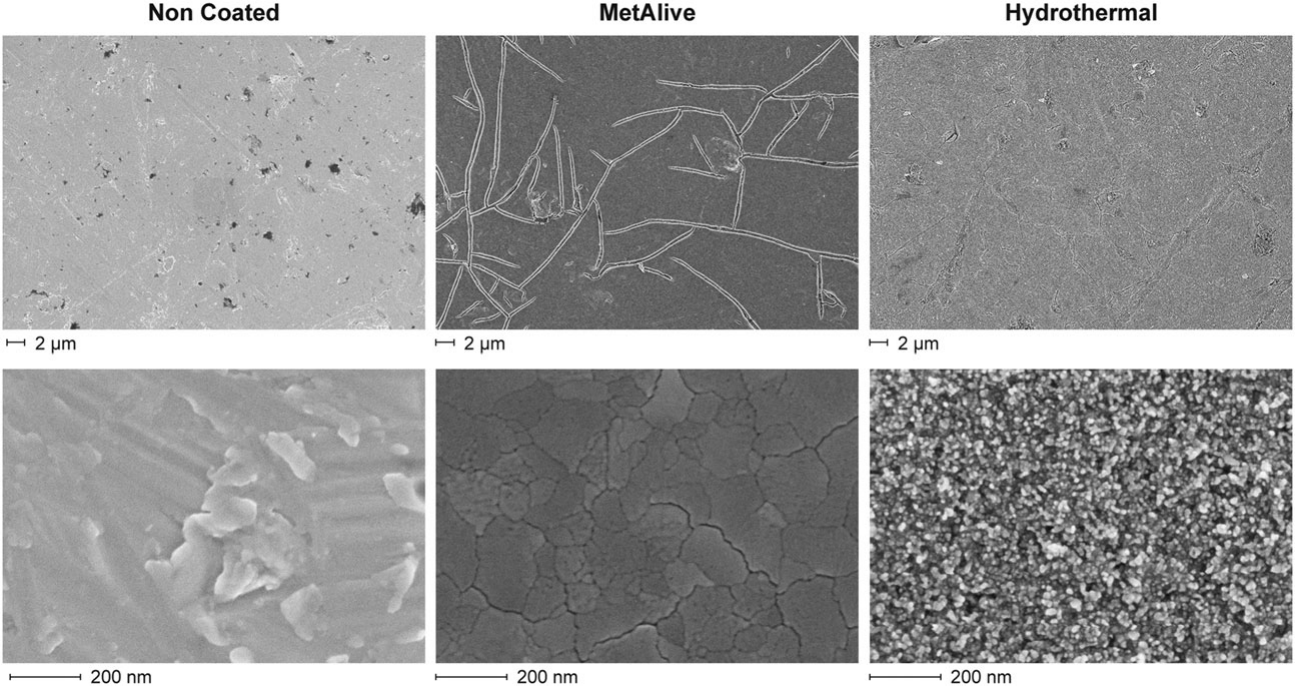
Scanning electron microscopy images of the substrate investigated show surfaces topography at low and high magnification

The SEM experiment was carried out to investigate the surface topography of the substrates. Figure 4 shows SEM images of the substrate surfaces at low and high magnification. The NC substrates showed a smooth surface with some grinding lines spreading over the surfaces. The MA surface showed a uniform smooth surface with extensive cracking, whereas the HT surfaces were fully covered with the coating crystals consisting of nearly spherical nanoparticles of 20–50 nm.

**Figure 5:**
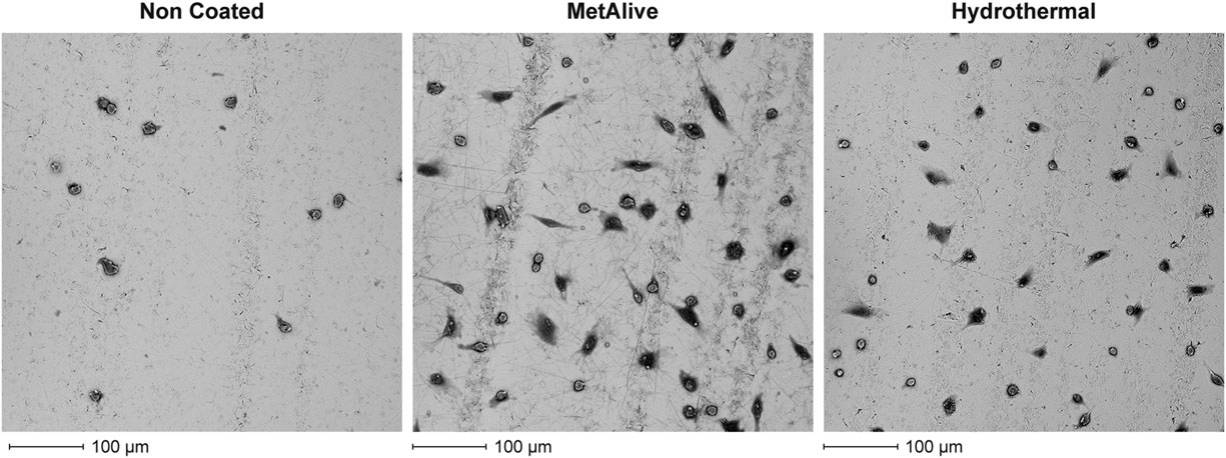
Scanning electron microscopy images of adherent fibroblasts on the substrate investigated. After 6 hr of adhesion followed by 15 min of trypsinization, less cells with a rounded shape were formed on the non‐coated substrate surface; the hydrothermal and MetAlive™ coating surfaces showed more cells with an elongated shape with extracellular fibrils extending towards the treated surface

**Figure 6:**
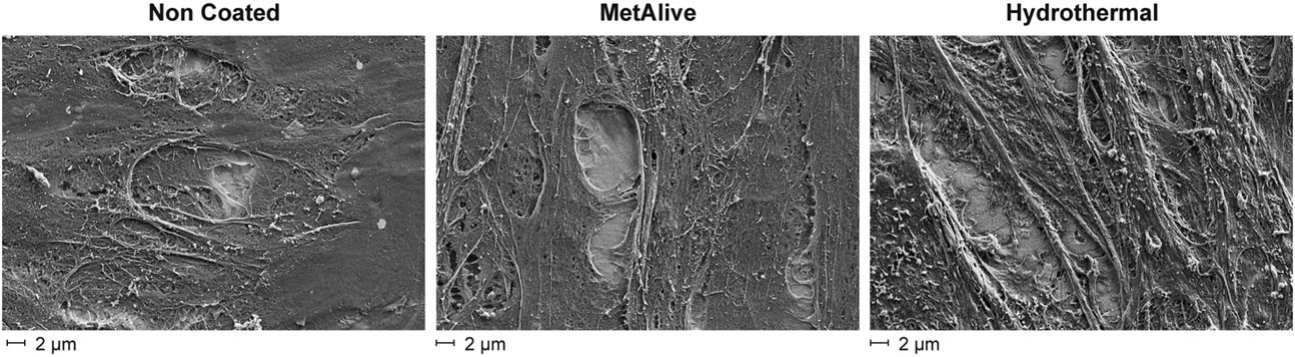
Proliferation results for ultraviolet‐treated substrates were confirmed with scanning electron microscopy images showing a thick and uniform cell mass at Day 10

SEM images of adherent fibroblasts on the substrate surfaces after 6 hr of adhesion followed by 15 min of trypsinization showed less cells and with a rounded shape on the NC substrate surfaces, whereas the HT and MA surfaces showed more cells with an elongated shape with extracellular fibrils extending towards the treated surface (Figure 5). No differences in the mode of cell adhesion between the HT and MA groups were noticed. Proliferation results for UV‐treated substrates were confirmed with SEM images showing a thick and uniform cell mass at Day 10 (Figure 6).

## 4 DISCUSSION

In this study, we evaluated the effects of oxidized nanostructured titanium surfaces on the interaction between treated titanium surfaces and human gingival fibroblasts in comparison with the non‐coated titanium surfaces. The wettability of a material has been considered as a predictive indicator of cytocompatibility. The differences among the groups we observed are related to the differences in surface topographies and crystalline structures of their outermost surfaces. These findings indicated that the hydrophilicity of the nanostructured TiO₂ surfaces was stronger than that of NC Ti‐6Al‐4V surfaces. After UV light treatment, all surfaces became remarkably more wettable to water; the contact angles decreased dramatically enhancing the hydrophilicity of the substrates. The UV‐treated nanostructured TiO₂ surfaces had a low contact angle, which indicated their superior wettability compared with the NC titanium surfaces. UV treatment converts surfaces from hydrophilic to super hydrophilic and cleans the contaminated hydrocarbons by reducing the carbon percentage to less than 20% (Aita et al., 2009).

Wettability is an important factor for cell adhesion as shown by numerous studies. It is believed that hydrophilic surfaces would enhance fibroblast attachment and spreading compared with hydrophobic surfaces (van Wachem et al., 1985; Webb, Hlady, & Tresco, 1998). Shi et al. (2015) observed that a moderately wettable TiN hydrothermally treated surface with a contact angle of around 40° can improve fibroblast adhesion (Shi et al., 2015). However, the role of the surface hydrophilicity in determining the biocompatibility of the material is controversial. There is no sufficient evidence to support the relationship between the degree of hydrophilicity and osseointegration capability of titanium implants (Ogawa, 2014). Several advanced modifications and techniques are available to obtain biologically active surfaces on the nanoscale level. These include physical approaches, chemical approaches, nanoparticle deposition, and self‐assembly of monolayers (Mendonca et al., 2008). The early cell adhesion to biomaterial surfaces relies on the protein adsorption that bind to the implant material surfaces and adheres by surface receptors forming focal adhesions (Areva et al., 2004). Under culture conditions, trypsin–EDTA can disturb these adhesions (Wilson, Clegg, Leavesley, & Pearcy, 2005), and rounded cells will detach from the surface upon gentle trypsinization. However, this method provides only indirect evidence about single cell's adhesion strength on solid surface. Real mechanical adhesion strength evaluation would require the use of equipment that facilitates single cell mechanical loading in several directions. In this study, we found a significant difference in the amount of adherent cells between the different substrate groups. Human gingival fibroblasts were more resistant to enzymatic detachments with trypsin–EDTA on MA and HT coated than on NC titanium substrates. This is in accordance with Guida et al. (2013), who demonstrated that gingival fibroblast functions were stimulated by oxidized nanostructured titanium surfaces. However, in our study, after the UV light treatment, the amount of attached cells on the MA, HT, or NC surfaces did not differ significantly. Many in vitro studies have revealed that after UV treatment, a remarkable enhancement of attachment and retention of osteogenic cells derived from animals and humans occurs (Aita et al., 2009; Hori et al., 2010). Aita et al. (2009) reported that UV‐treated titanium implants up to 48 hr showed three times greater strength of osseointegration compared with untreated implants leading to complete establishment of osseointegration with nearly 100% bone to implant contact as opposed to less than 55% for untreated implants (Aita et al., 2009). Saita et al. (2016) showed that the attachment, spreading, proliferation, and alkaline phosphate activity of bone marrow‐derived osteoblasts were enhanced on Ti or apatite‐coated Ti specimens after photofunctionalization for 20 min compared with non‐treated specimens (Saita et al., 2016). Furthermore, Yamada, Yamada, Ueda, and Sakurai (2014) investigated that UV irradiation for 8 hr reduced the attachment and biofilm formation of wound pathogens on various topographical Ti surfaces (Yamada et al., 2014). Varying UV treatment times have been used in previous studies. In our study, 15 min UV treatment seemed to be sufficient to enhance surface wettability but may not be optimal to improve cell behavior. Different UV treatment times may be required for optimizing the conditions for diverse biologic responses. Our SEM analysis showed that fibroblast cells had intimate spreading on the substrate surfaces. Elongated cells with many cellular fibrils were seen on all substrates. However, after the detachment assay, the difference in cell adherence was visible where less cells with a more rounded shape were seen on the NC than on the HT or MA surfaces. All substrate types showed an increase in proliferation rate until 10 days. Non‐UV‐treated substrates showed significant differences in cell activities among the materials in all time points, proliferation rates being higher on NC than on HT surfaces. Our findings might be explained by the fact that cells simply attach better on the HT or MA and UV‐treated surfaces, which may slow down the proliferation rate.

Several studies proposed that implant surface characteristics of the transmucosal area can influence the attachment of peri‐implant mucosal tissue, and certain surface roughness may promote soft tissue sealing, increasing the interaction between implant surfaces and soft tissue attachment (Schupbach & Glauser, 2007; Welander et al., 2008). Good soft tissue attachment around the dental implant is established during the early stages of wound healing. Our findings indicate that both MA and HT surfaces with UV surface treatment may promote early stages of implant wound healing. This, however, calls for further blood compatibility and protein adhesion investigation, and final performance has to be evaluated in clinical conditions, which will be the focus of our future studies.

In conclusion, this *in vitro study* demonstrated that nanostructured titanium oxide implant surfaces, obtained by sol–gel and HT coating methods, enhance the surface wettability and encourage human gingival fibroblast adhesion and proliferation when compared with non‐coated surfaces. UV light treatment clearly improves the wettability of all examined Ti‐6Al‐4V surfaces.

## CONFLICT OF INTEREST

None declared.
